# Multidisciplinary Strategies to Manage Treatment-Related Dermatologic Toxicity and Improve Quality of Life in Cancer Patients: A Narrative Review

**DOI:** 10.3390/healthcare14162514

**Published:** 2026-08-12

**Authors:** Joaquim Faria Monteiro, Clémence Salvador-Entradas, Dorian Marrot, Maribel Teixeira, Nuno Milhazes, Vera Almeida, Ana Teixeira

**Affiliations:** 1 UCIBIO, Applied Molecular Biosciences Unit, i4HB, Faculty of Pharmacy, University of Porto, Rua de Jorge Viterbo Ferreira, No. 228, 4050-313 Porto, Portugal; j.monteiro@ff.up.pt (J.F.M.); ana.teixeira@iucs.cespu.pt (A.T.); 2 Laboratory of Pharmacology, Department of Drug Sciences, Faculty of Pharmacy, University of Porto, Rua de Jorge Viterbo Ferreira, No. 228, 4050-313 Porto, Portugal; 3 University Institute of Health Sciences IUCS, CESPU, CRL, 4585-116 Gandra, Portugal; a34063@alunos.cespu.pt (C.S.-E.); a34036@alunos.cespu.pt (D.M.); 4 Associate Laboratory i4HB, Institute for Health and Bioeconomy, University Institute of Health Sciences, CESPU, 4585-116 Gandra, Portugal; maribel.teixeira@iucs.cespu.pt (M.T.); nuno.milhazes@iucs.cespu.pt (N.M.); 5 UCIBIO, Applied Molecular Biosciences Unit, Translational Toxicology Research Laboratory, University Institute of Health Sciences (1H-TOXRUN, IUCS-CESPU), 4585-116 Gandra, Portugal; 6 UNIPRO, Research Unit in Oral Pathology and Rehabilitation, University Institute of Health Sciences (IUCS), CESPU, 4585-116 Gandra, Portugal; 7 MEDTECH-Pharmaceutical Technology Laboratory, Faculty of Pharmacy, University of Porto, Rua de Jorge Viterbo Ferreira 228, 4050-313 Porto, Portugal

**Keywords:** pharmaceutical care, cancer treatment, dermatologic toxicity, adherence to treatment, quality of life, body image

## Abstract

**Highlights:**

**What are the main findings?**

- Dermatologic toxicities are highly prevalent across anticancer therapies and significantly impact patients’ quality of life, body image, and treatment adherence.
- Proactive and multidisciplinary management—particularly involving pharmaceutical care—can reduce severity of toxicities and promote treatment adherence, despite the certainty of the evidence being low to moderate.

**What are the implications of the main findings?**

- Early identification and structured management of dermatologic adverse events should be integrated into routine oncology care to improve patient outcomes.
- There is a need for standardized, evidence-based pharmaceutical care protocols and higher-quality studies to guide clinical practice in oncodermatology.

**Abstract:**

Background/Objectives: Dermatologic toxicities are among the most common adverse effects of anticancer therapies, including chemotherapy, targeted therapies, and immunotherapies. Although typically non-life-threatening, these conditions can significantly impair quality of life, alter body image, and lead to treatment non-adherence which may compromise health outcomes. This systematic review aims to synthesize the evidence on the incidence, severity, and management of treatment-related dermatologic toxicities, with a focus on pharmaceutical and multidisciplinary care strategies. Methods: This review followed the PRISMA 2020 guidelines. PubMed, ScienceDirect, and Cochrane were searched for studies published between 1 January 2015 and 31 December 2025. Eligibility criteria were defined using the PICO (R) framework. Study selection, data extraction, and risk-of-bias assessment were performed independently by two reviewers. Given study heterogeneity, a narrative synthesis was conducted. Results: Seventeen studies were included. Dermatologic toxicities were frequently reported during cancer treatment and were generally mild to moderate in severity, although they imposed a substantial burden on patients. Supportive dermatologic care strategies were associated with reduced symptom severity and improved quality of life in some studies. Evidence for pharmaceutical care and multidisciplinary management was more limited, and the available data do not allow firm conclusions regarding their effectiveness. Conclusions: Dermatologic toxicities remain an important challenge in oncological care. The evidence suggests potential benefits of supportive care approaches, but the certainty of the evidence is limited, particularly for pharmaceutical care. Further high-quality studies are needed to clarify the impact of these interventions and support standardized recommendations.

## 1. Introduction

Cancer remains the second leading cause of death worldwide and is among the leading causes of premature mortality in most countries, accounting for nearly 10 million deaths annually [1]. Patients undergoing anticancer therapy frequently experience treatment-related adverse effects, including dermatological toxicity reactions such as alopecia, xerosis, pruritus, and changes in skin pigmentation [2]. These cutaneous adverse effects (CAEs) extend beyond physical discomfort, significantly affecting body image perception, impairing quality of life, and contributing to psychological distress, reduced self-esteem, and social stigmatization [3,4]. Adjustment of anticancer therapy, dose reduction, or discontinuation due to dermatologic toxicity may compromise treatment effectiveness and can negatively impact clinical outcomes, highlighting the need for early prevention and proactive management strategies [5].

Approaches with a multidisciplinary framework should be considered a key strategy in the management of these toxicities, and pharmacists are strategically integrated within oncology care pathways and can contribute to the appropriate balance between treatment efficacy and tolerability, thereby supporting the optimization of therapeutic outcomes and the management of adverse events [6]. Pharmacists can provide structured medication review, early identification of treatment-related toxicities, patient education, adherence support, and timely referral when problems arise. Growing evidence suggests that pharmaceutical care interventions improve treatment-related symptom control, reduce toxicity, and enhance adherence, supporting the implementation of structured services in community pharmacy settings [7,8,9,10].

Multidimensional therapeutic approaches that integrate both physical and psychological components, delivered by multidisciplinary care teams, have demonstrated improvements in treatment outcomes and health related quality of life in cancer populations [11]. However, research specifically assessing the psychological sequelae of dermatologic toxicities is scarce, and evidence based psychoeducational interventions tailored to cancer patients experiencing skin related side effects remains limited [12,13,14]. Recent findings have highlighted even more the association between dermatologic side effects and negative alterations in body image, with significant impacts on mental health and overall quality of life [15,16], underscoring the overall psychosocial burden and the need for targeted supportive care strategies to mitigate psychosocial distress and optimize quality of life among cancer survivors.

Currently, no standardized multidisciplinary approach has been established for the management of dermatologic toxicities, including interventions involving pharmacists and psychologists. This review aimed to synthesize the available evidence on the incidence, severity, and management of treatment-related dermatologic toxicities in cancer patients, with particular attention to supportive, pharmaceutical, and multidisciplinary care strategies. The principal novelty is that it brings together primary studies, narrative and systematic reviews, and clinical guidelines in one implementation-focused synthesis to identify what is directly supported by evidence, where the literature remains sparse, and which practical management elements still rely mainly on indirect or expert-opinion evidence.

## 2. Materials and Methods

### 2.1. Protocol and Registration

This review was designed and reported in accordance with the Preferred Reporting Items for Systematic Reviews and Meta-Analyses (PRISMA 2020) statement [17], the completed PRISMA 2020 checklist is provided in Supplementary File S.1. Because the synthesis was qualitative, reporting was additionally informed by the Synthesis Without Meta-analysis (SWiM) guideline.

The protocol was prospectively registered in the International Prospective Register of Systematic Reviews (PROSPERO; registration number CRD420261379647, available from https://www.crd.york.ac.uk/PROSPERO/view/CRD420261379647). No amendments were made to the registered protocol during the conduct of the review.

The review question and eligibility criteria were prospectively defined using the PICO (R) framework.

### 2.2. Research Question and Eligibility Criteria

The research question was formulated as follows: “In oncology patients receiving anticancer therapy, what is the reported incidence and management of treatment-related dermatologic toxicities, and what multidisciplinary care strategies have been implemented to prevent, monitor, or manage these toxicities effectively, thereby supporting treatment adherence and quality of life?”

The PICO (R) components were defined as follows:

Population (P): Oncology patients receiving anticancer therapy who are at risk for or experience treatment-related dermatologic toxicity.

Intervention (I): Preventive, monitoring, or management interventions, pharmaceutical or multidisciplinary, targeting treatment-related dermatologic toxicities.

Comparison (C): Usual care or absence of structured intervention.

Outcomes (O): Incidence, severity, or evolution of dermatologic toxicity; effectiveness of management strategies; treatment adherence; and health-related quality of life (including body image outcomes).

Context (R): Hospital, oncology, or pharmacy-based care settings.

Inclusion criteria were:

- Studies reporting on the incidence or prevalence of dermatologic toxicities in oncology patients undergoing anticancer therapy OR
- Studies evaluating preventive, monitoring, or management strategies for dermatologic toxicities, including pharmaceutical and multidisciplinary care interventions OR
- Studies addressing patient-reported outcomes (such as body image or quality of life) in the context of dermatologic toxicity AND
- Study designs including randomized or non-randomized clinical trials, observational studies, systematic or narrative reviews, and clinical practice guidelines AND
- Publications in English, Portuguese, Spanish, or French within the last 10 years.

Exclusion criteria were:

- Single case reports, editorials, commentaries, and letters to the editor OR
- Non-human studies.

### 2.3. Information Sources and Search Strategy

A systematic search of three electronic databases—PubMed (MEDLINE), ScienceDirect (Elsevier), and the Cochrane Central Register of Controlled Trials—was conducted between 1 January 2015 and 31 December 2025. The databases were selected to provide broad coverage of the relevant literature, combining extensive biomedical indexing, full-text clinical and pharmaceutical research, and high-quality evidence syntheses. Together, these sources were considered sufficient to identify studies on treatment-related dermatologic toxicities and their management.

The search strategy combined Medical Subject Headings (MeSH) and free-text keywords related to three conceptual blocks—oncology population, dermatologic toxicity, and pharmaceutical or supportive care—combined with Boolean operators (AND, OR), including but not limited to: “oncology patients”, “cancer therapy”, “dermatologic toxicity”, “cutaneous adverse effects”, “skin toxicity”, “pharmaceutical care”, “clinical pharmacy”, “body image”, and “quality of life”.

Database-specific search strategies were developed and refined iteratively. Filters were applied to limit results to human studies and the languages specified above. For each database, the full electronic search strategy (including all search terms, Boolean operators, and limits) is documented in Table 1, in line with PRISMA 2020 recommendations. The date of the last search was December 2025. Reference lists of relevant reviews and included articles were hand-searched to identify additional eligible studies.

### 2.4. Study Selection

All records retrieved from the searches were imported into EndNote, and duplicates were removed automatically and checked manually. Study selection occurred in two stages. First, two reviewers independently screened titles and abstracts to identify potentially eligible studies. Second, full texts of the selected records were assessed independently against the predefined inclusion and exclusion criteria.

To ensure consistency, the same eligibility criteria and a standardized screening form were applied throughout the process, and any uncertainties were discussed between reviewers before a final decision was made.

Disagreements at any stage were resolved through discussion and, if necessary, consultation with a third reviewer. The study selection process was documented using a PRISMA 2020 flow diagram, including numbers of identified, screened, excluded, and included records and the main reasons for exclusion at the full-text stage.

### 2.5. Data Extraction

Data from the included studies were extracted using a standardized, piloted data extraction form. The following information was collected:

- Study characteristics: first author, year of publication, country, setting, and study design.
- Population: sample size, demographic and clinical characteristics, cancer type, and anticancer treatments.
- Intervention: description of the pharmaceutical care or multidisciplinary intervention (content, timing, duration, frequency, setting, and professionals involved).
- Comparators: description of usual care or alternative interventions.
- Outcomes: measures related to dermatologic toxicity (incidence, severity, grading, time to onset), treatment adherence, health-related quality of life, and body image.
- Key findings and authors’ conclusions.
- Reported limitations and potential sources of bias.

Two reviewers performed data extraction independently. Any discrepancies were solved by consensus, with arbitration by a third reviewer.

### 2.6. Risk of Bias and Quality Assessment

Methodological quality and risk of bias were assessed independently at the study level by two reviewers, using tools selected according to study design. Controlled and uncontrolled clinical trials were appraised with the Cochrane Risk of Bias 2 (RoB 2) tool when randomized, and with ROBINS-I when non-randomized. Controlled and uncontrolled observational studies were assessed using appropriate critical appraisal checklists (such as Newcastle–Ottawa Scale) adapted to the study design and methodological features, with particular attention to selection bias, confounding, exposure measurement, outcome assessment, and completeness of follow-up. Systematic reviews were evaluated with ROBIS to assess risk of bias in the review process and with AMSTAR 2 to appraise methodological quality. Narrative reviews were not subjected to formal quantitative risk-of-bias scoring but were critically examined for transparency of search strategy, selection methods, evidence synthesis, and potential for interpretive bias. The overall certainty of evidence for key outcomes was summarized using GRADE, considering risk of bias, inconsistency, indirectness, imprecision, and publication bias. Any disagreements between reviewers were resolved by discussion and, when necessary, by consultation with a third reviewer.

### 2.7. Data Synthesis

Given the anticipated heterogeneity in study designs, interventions, and outcome measures, a primarily narrative synthesis was conducted. Primary evidence, including clinical trials and observational studies, was identified and cross-checked against evidence synthesized in reviews and guidelines to avoid duplication of information. When a topic was supported by both primary studies and secondary reviews, the primary studies were prioritized for outcome synthesis, and the reviews were used only for background and contextual interpretation. We included both primary studies and secondary sources because the literature specifically evaluating pharmaceutical care is limited and heterogeneous and combining them allowed us to capture direct outcomes while also situating the findings within the broader clinical and methodological context.

Studies were grouped and described according to:

- Type and severity of dermatologic toxicity.
- Type and setting of pharmaceutical care intervention (e.g., preventive skin-care counselling, monitoring and early management of cutaneous events, adherence support, structured toxicity management protocols).
- Reported effects on toxicity outcomes, treatment adherence, quality of life, and body image.

## 3. Results

The complete PRISMA checklist (Supplementary File S.1) is provided as Supplementary Materials.

**Figure 1:**
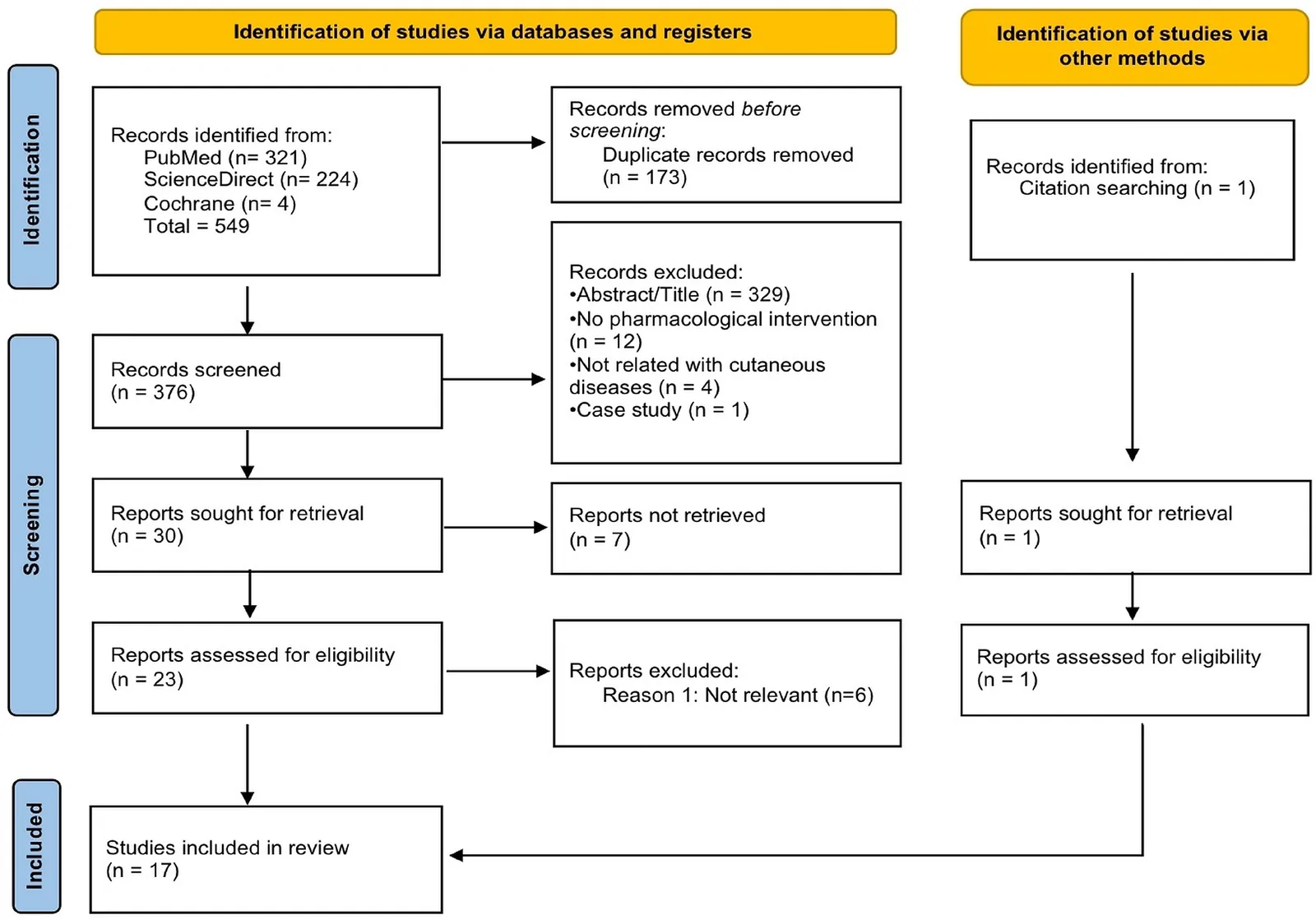
PRISMA 2020 flow diagram.

Figure 1 presents the PRISMA 2020 flow diagram, illustrating the study selection process—from the initial records identified through database searches and other sources, to screening by title and abstract, full-text assessment, exclusions with reasons, and the final number of studies included in the qualitative synthesis.

The study selection process is summarized in a PRISMA 2020 flow diagram. A total of 549 records were identified through database searching, with 173 duplicates removed before screening; after title/abstract screening, 30 reports were sought for retrieval, 7 could not be retrieved, and 23 reports were assessed for eligibility, of which 6 were excluded as not relevant (irrelevance to cutaneous diseases or case study design). One additional record was identified through citation searching, and the review ultimately included 17 studies. The included evidence comprised 2 randomized controlled trials, 7 non-randomized interventional/observational studies, and 5 reviews (1 systematic and 4 narrative reviews).

This narrative review was structured based on the selected studies and is presented in three main sections. Initially, the review examines three chemotherapy categories (chemotherapy, targeted therapy and immunotherapy, highlighting the incidence and severity of dermatologic adverse events. It then discusses the principal findings on event management, including the assessment of risk of bias and certainty of evidence. Finally, it provides a synthesis of the adverse events identified and proposes a management strategy for oncology patients.

Supplementary File S.2 summarizes the characteristics of the included studies, including study design, population, intervention, comparators, and outcomes. This table offers a concise overview to facilitate comparison across studies.

Table 2 and Table 3 details the main findings from the included studies (clinical and observational studies and reviews, respectively), alongside their methodological limitations, risk of bias assessments. Table 4 summarizes the GRADE assessment for the main outcomes addressed in this review. It highlights key results, sources of uncertainty (such as imprecision or inconsistency), and the overall strength of evidence supporting our discussion.

### 3.1. Chemotherapy-Related Toxicities

Chemotherapy-related dermatologic toxicities were reported in three primary studies and one secondary study, comprising one prospective observational cohort, two non-randomized observational studies, and one narrative review [21,24,25,33]. Across these studies, the most frequently described events were alopecia, xerosis, hand–foot syndrome, melanonychia, nail and mucosal toxicities, with taxanes, paclitaxel, docetaxel, and capecitabine emerging as the main implicated agents; in the prospective cohort by Anoop et al., CAEs were common and generally of low-to-moderate severity, although associated with relevant morbidity and quality-of-life burden [21]. Nail toxicities were also emphasized in the narrative review by Emvalomati et al., which identified chemotherapy, particularly taxanes, as a major contributor to matrix, bed, and periungual disorders, while suggesting that early recognition and management may improve adherence [33].

Evidence on management was mainly supportive and observational rather than comparative. Two observational studies evaluating dermocosmetic regimens during chemotherapy with or without radiotherapy suggested better tolerability, less worsening of skin toxicity, and improved perceived benefit, but both lacked robust control groups and were graded as very low-certainty evidence [24,25]. Overall, chemotherapy-related toxicities appeared mostly manageable with proactive skin barrier care, symptom-directed treatment, and early detection, although high-quality interventional data remain limited.

### 3.2. Targeted Therapy-Associated Reactions

Targeted therapy-associated dermatologic reactions were described in seven studies, including five primary studies and two secondary studies, covering retrospective cohorts, a prospective substudy, and narrative or systematic reviews [18,19,20,28,31,32,34]. EGFR inhibitors were consistently associated with acneiform or papulopustular eruptions and eczema, with reported incidence ranging from 50% to 90% in the narrative review by Macdonald et al. [34], while Kale et al. [18] reported an overall CAE incidence of 59%, predominantly acneiform rash and eczema, usually of low-to-moderate severity. MEK inhibitors were associated with a distinct toxicity profile, especially in pediatric populations, in whom xerosis, dermatitis, paronychia, and hair changes were more common than the acneiform rash typically described in adults; these toxicities were generally grade 1–2 but could still generate substantial clinical burden and health care use [19,20]. In patients receiving BTK inhibitors, Bitar et al. described frequent hair and nail toxicities, mostly grade 1–2, with a delayed onset and meaningful quality-of-life impact [28].

Management strategies for targeted therapy-related reactions were predominantly based on supportive and symptom-guided care. Narrative evidence suggested that topical and systemic treatments, sun protection, and early dermatology involvement may reduce symptom burden and help prevent treatment interruption, while in psoriasis-related reactions associated with EGFR inhibitors, TKIs, or ICIs, topical therapy, ultraviolet-based approaches, and acitretin were described as potential options in selected cases [31,32,34]. However, apart from sorafenib-associated HFSR, robust interventional evidence was scarce, and most conclusions for targeted therapies relied on observational data or low-certainty reviews.

### 3.3. Immune Checkpoint Inhibitor-Related Adverse Events

ICI-related dermatologic adverse events were addressed in five studies, including three primary studies and two secondary studies, consisting of one prospective cohort, two retrospective series, one narrative review, and one review focused on mechanistic and clinical patterns [22,26,27,30,31]. These studies identified cutaneous adverse events as among the most common immune-related adverse events, with eczema, morbilliform eruptions, acneiform reactions, vitiligo, pruritus, xerosis, lichenoid eruptions, folliculitis, and psoriasis exacerbation among the principal manifestations. In Keiser et al. [22], eczema was the most prevalent phenotype in patients receiving PD-1/PD-L1 inhibitors, with a median onset of around three months, and approximately 70% of cases resolved or improved with treatment; dose modification occurred in 13%, and discontinuation in 6%. Dika et al. [26] reported mostly mild early CAEs, although severe grade 4 reactions were rare but possible, and Shi et al. [27] described lichenoid eruptions as typically manageable and only rarely leading to discontinuation.

Management of ICI-related toxicities was largely based on topical corticosteroids, oral therapies in selected cases, and close dermatology-oncology collaboration. The mechanistic review by Eshaq et al. [30] emphasized that these reactions are biologically heterogeneous and may include vitiligo as a potential surrogate of response, whereas Madan et al. [31] suggested that early multidisciplinary management may help reduce treatment interruption in psoriasis-related presentations, although the supporting evidence was low. Taken together, the available studies indicate that most ICI-related dermatologic adverse events are mild to moderate and manageable with supportive care, but serious reactions can occur and require prompt recognition and individualized intervention.

The findings of the included studies highlight that dermatologic adverse events are frequent across several anticancer regimens, ranging from common low-grade toxicities, such as xerosis, pruritus, and acneiform eruptions, to less frequent but clinically significant complications, including hand–foot syndrome, radiation dermatitis, paronychia, and severe cutaneous adverse reactions. Although the severity of these events is often mild to moderate, their cumulative impact on discomfort, cosmetic appearance, functional capacity, and treatment adherence can be substantial. Across the reviewed studies, early recognition, structured patient education, and prompt initiation of supportive skin care consistently emerged as the most important measures to mitigate symptom burden and preserve oncologic treatment continuity.

Evidence from the review supports a management approach tailored to the type and severity of toxicity. For acneiform eruptions and folliculitis, the literature favors gentle skin care, avoidance of irritants, topical corticosteroids, and tetracycline-class antibiotics in selected cases, with the strongest support coming from observational studies and clinical guidance, while randomized evidence remains limited. In hand–foot syndrome, prophylactic urea-based creams showed the most consistent benefit, including randomized data demonstrating reductions in early toxicity and improvement in quality of life, whereas other preventive strategies were supported mainly by lower-level evidence. For radiation dermatitis, basic preventive measures such as moisturization, friction avoidance, and skin education were better supported than more advanced topical regimens. Xerosis, pruritus, pigmentary changes, alopecia, and nail toxicity were also frequent, but the available evidence was largely observational or expert based, reinforcing the importance of individualized supportive care rather than uniform pharmacologic intervention.

Overall, the studies included in this review indicate that effective management of dermatologic adverse events should be proactive, preventive, and multidisciplinary. While the certainty of evidence varies across toxicities, the consistent message is that early intervention may reduce symptom severity, improve quality of life, and support treatment adherence in oncology patients.

Table 5 presents a practical synthesis of the management strategies for the main dermatologic adverse events identified in this review. This table was developed by the study team based on the findings of the included studies and integrates the available evidence on preventive and therapeutic approaches according to the type of toxicity. Its purpose is to provide a clinically oriented summary to support the recognition and management of these adverse events in oncology patients.

## 4. Discussion

The initial objective of this review was to synthesize the evidence of pharmaceutical interventions aimed at mitigating the dermatological side effects of cancer treatment. However, the scarcity of studies specifically evaluating such interventions or their impact precluded a focused synthesis. Consequently, the scope was broadened to a comprehensive synthesis of the current evidence on the incidence and management of CAEs in cancer patients, within which the role of pharmaceutical and multidisciplinary care was interpreted.

### 4.1. Interpretation in the Context of Previous Evidence

Our findings suggest that CAEs are highly prevalent across chemotherapy, targeted therapies, and immunotherapies, and appear to impact treatment adherence, psychological well-being, and clinical outcomes [35,36]. Targeted therapies, particularly EGFR inhibitors and multikinase inhibitors, were frequently associated with acneiform rash, xerosis, pruritus, paronychia, and hand–foot skin reaction (HFSR) [37,38]. These toxicities are largely mechanism-based and, in some cases, correlate with therapeutic efficacy, reinforcing their potential role as surrogate biomarkers [39]. In contrast, immune checkpoint inhibitors (ICIs) predominantly induced immune-mediated manifestations, including eczematous, morbilliform, and lichenoid eruptions, as well as vitiligo and psoriasis exacerbation. These findings reflect immune dysregulation and are consistent with observational evidence linking dermatologic immune-related adverse events to improved clinical outcomes. Chemotherapy-related toxicities, such as alopecia, hyperpigmentation, nail changes, and xerosis [25,32], although less mechanistically specific, remain frequent and contribute substantially to the overall patient burden [40,41,42].

### 4.2. Severity, Patient Burden and Effectiveness of Supportive Interventions

Most identified CAEs were mild to moderate and rarely life-threatening. However, their high frequency, persistence and visibility contribute to a significant burden on patients, reducing well-being, and reinforcing their multidimensional impact on patients’ quality of life. Even non-severe toxicities may lead to non-adherence to treatment (dose reductions or treatment interruptions), potentially compromising treatment efficacy. The clinical relevance of CAEs is not directly linked with the physical severity of these effects and should incorporate patient-centered impact [43].

Targeted dermatologic interventions, including topical corticosteroids, antibiotics, emollients, and barrier-repair formulations, have been reported to reduce the severity of CAEs when implemented early [2,44], although most supporting evidence derives from observational studies with low to moderate certainty. These strategies reflect a shift from reactive to preventive care, emphasizing the importance of anticipating toxicity based on treatment type and individual risk profiles. Evidence from multiple studies supports the prophylactic use of dermatologic formulations, such as urea-based, to reduce severity and delay the onset of HFSR without compromising treatment efficacy. In patients receiving EGFR-targeted therapies, such approaches have also been associated with reduced toxicity severity and improved treatment continuity [45,46]. Similarly, structured skincare protocols aimed at preserving skin barrier function have been linked to delayed onset and decreased severity of radiotherapy and drug-induced skin reactions [47].

### 4.3. Role of Pharmaceutical and Multidisciplinary Care

Proactive dermatological interventions, including education on skincare and the use of tailored dermocosmetics, can reduce the severity of CAEs and enhance the patient experience. In this context, integrating dermatological management into standard oncology protocols is important to minimize the CAEs, promote treatment adherence and optimize therapeutic outcomes [41,48]. Pharmaceutical interventions can, in this context, have significant implications for improving patients’ quality of life and clinical treatment outcomes. Dermatologic toxicities often carry a visible and psychosocial burden, adversely affecting body image, emotional well-being, and social functioning. Effective management therefore extends beyond symptom control to encompass a more holistic, patient-centered approach that addresses both the physical and psychological dimensions of care. By reducing discomfort, improving self-perception, well-implemented pharmaceutical interventions contribute to a more tolerable cancer experience and reinforce the importance of supportive care as a core component of modern oncology [49]. However, evidence supporting broader preventive strategies, such as dermocosmetic regimens, remains limited and is largely derived from observational studies.

Pharmacists can also identify potential drug-related causes of dermatologic toxicity, optimize medication regimens, and prevent drug–drug interactions that may exacerbate CAEs. Variability in dermatologic toxicity profiles has been associated with pharmacokinetic and pharmacogenomic factors, particularly in patients treated with tyrosine kinase inhibitors [50]. This suggests that biomarker-driven strategies could improve the prediction and prevention of adverse events, enabling more individualized supportive care, though findings require validation in larger prospective cohorts. However, current evidence remains limited, indicating a need for further research into predictive models and personalized interventions.

Another finding of this review is the apparent relevance of multidisciplinary management, essential for accurate diagnosis, early detection, and tailored management of dermatologic toxicities [51]. This collaborative approach has been consistently recommended, particularly given the complexity and heterogeneity of skin toxicities associated with recent anticancer therapies. Furthermore, multidisciplinary care enhances patient education and engagement, which are critical for adherence to both oncologic and supportive treatments.

Despite these advances, several limitations in the existing literature should be acknowledged. There is a lack of large-scale randomized controlled trials specifically addressing the management of dermatologic toxicities, with many recommendations still based on expert consensus or observational studies. Additionally, heterogeneity in outcome measures, particularly regarding quality of life, limits comparability across studies. Certain areas, such as nail toxicities and long-term dermatologic sequelae, remain underexplored despite their relevance to patient well-being. These gaps highlight the need for more rigorous and standardized research in oncodermatology.

## 5. Limitations and Future Research

This review presents limitations that should be acknowledged; the heterogeneity of study designs, interventions, and outcome measures precluded quantitative synthesis and limited comparability across studies. The exclusion of additional databases and clinical trial registries may have limited the comprehensiveness of the search and the possibility of identifying relevant studies indexed elsewhere or unpublished evidence. The overall certainty of evidence is limited by the predominance of retrospective cohorts, small sample sizes, and single-center designs. Furthermore, patient-reported outcomes and long-term follow-up are often lacking, and narrative reviews and case series contribute to lower levels of evidence. These limitations underscore the need for well-designed prospective and randomized studies. The absence of standardized reporting of dermatologic outcomes across studies further constrained the consistency and interpretation of results. Additionally, the inclusion of studies with predominantly low to moderate methodological quality and potential risk of bias may affect the robustness of the findings.

Future research and practice should focus on systematically translating the emerging evidence into structured models of pharmaceutical care for oncology patients with dermatologic toxicity. Priority areas include the definition of core components for assessment and monitoring that can be realistically implemented in both hospital and community pharmacy settings, ensuring early identification and consistent grading of CAEs. There is also a need to consolidate and test those interventions that show the most robust and consistent benefits in reducing dermatologic toxicity and sustaining adherence to anticancer therapy, while preserving patients’ quality of life.

In parallel, future work should evaluate the feasibility, resource requirements, and organizational impact of integrating structured skin toxicity management pathways into routine oncologic care, including training needs for pharmacists and collaborative workflows with oncology and dermatology teams. Collectively, these developments may provide a solid foundation for the design, piloting, and refinement of evidence-based protocols aimed at optimizing pharmaceutical care for cancer patients experiencing treatment-related dermatologic toxicities.

## 6. Conclusions

The development of standardized, evidence-based guidelines for the prevention and management of treatment-related dermatologic toxicities in cancer patients remains an important goal to help optimize therapeutic outcomes and minimize adverse effects. As anticancer therapies continue to evolve, particularly with the growing use of targeted and immunotherapies, dermatological toxicities are likely to become more prevalent and complex, which underscores the potential value of proactive and multidisciplinary care approaches. The available evidence, largely observational and of low to moderate certainty, suggests that supportive and multidisciplinary care contributes to reducing the burden of dermatologic toxicity; however, given these methodological limitations, causal inferences cannot be drawn, and stronger comparative and prospective studies are needed before standardized pharmaceutical care protocols can be recommended.

## Figures and Tables

**Table 1:** Database search strategies used in the literature search.

Database	Search Strategy	Limits Applied
PubMed	1. (“chemotherapy” OR “cancer treatment”) AND (“skin side effect” OR “cutaneous toxicity”) AND “antineoplastic” 2. “cutaneous adverse effects” AND “oncology” 3. “cancer therapy” AND “dermatologic toxicity” 4. (“chemotherapy” OR “cancer treatment”) AND (“skin side effect” OR “cutaneous toxicity”) AND “frequency” 5. (“dermatologic toxicity” OR “cutaneous adverse effects”) AND (“cancer therapy” OR “oncology patients”) 6. (“chemotherapy” OR “cancer treatment”) AND (“skin side effect” OR “cutaneous toxicity”) AND “antineoplastic” AND (“treatment” OR “management”)	Publication date restricted to the last 10 years
ScienceDirect	1. oncology patients AND (dermatologic toxicity OR cutaneous adverse effects) AND (pharmaceutical care OR management) 2. oncology patients AND (dermatologic toxicity OR cutaneous adverse effects) AND (pharmaceutical care OR management) AND skin care	Publication date restricted to the last 10 years; search fields limited to title, abstract, and keywords; additional terms included cancer, cutaneous side effect, management, and skin care
Cochrane Library	MeSH descriptor: [Antineoplastic Agents] AND MeSH descriptor: [Skin] explode all trees AND MeSH descriptor: [Drug-Related Side Effects and Adverse Reactions]	Publication date restricted to the last 10 years

**Table 2:** Main findings of the included clinical and observational studies and risk of bias assessments and GRADE evaluation.

Reference (Year)	Main Finding	Risk of Bias
Kale et al. (2025) [18]	Cutaneous adverse events occurred in 59% of patients, were mainly acneiform eruptions and eczema related to EGFR inhibitors and were usually manageable without treatment discontinuation.	Moderate (ROBINS-I: with concerns mainly related to confounding and selection bias).
Friedland et al. (2024) [19]	Pediatric MEK inhibitor therapy was associated with frequent mainly mild dermatologic toxicities, particularly xerosis, dermatitis, paronychia, and hair changes, although these still caused clinical burden.	Moderate (ROBINS-I: with moderate concerns for confounding and outcome assessment).
Dávila Osorio et al. (2021) [20]	Pediatric patients receiving MEK inhibitors showed universal cutaneous toxicity, with eczema, hair changes, paronychia, and acneiform manifestations differing from adult patterns.	High (ROBINS-I: with moderate-high confounding and selection bias in a single-center retrospective chart review).
Anoop et al. (2021) [21]	Chemotherapy-related toxicities, including alopecia, xerosis, hand–foot syndrome, and melanonychia, were common and mainly associated with taxanes, paclitaxel, docetaxel, and capecitabine.	Moderate (ROBINS-I: with concerns related to selection and outcome assessment).
Keiser et al. (2021) [22]	Immune checkpoint inhibitor-related skin toxicities were most often eczematous or morbilliform, and most cases improved or resolved with topical or oral treatment, with limited treatment discontinuation.	Moderate-high (ROBINS-I: with high concerns for selection bias and confounding).
Lee et al. (2020) [23]	Prophylactic 20% urea cream did not reduce overall 12-week HFSR incidence but reduced early grade II or higher HFSR and improved quality of life in sorafenib-treated patients.	Low-moderate (RoB-2: randomized double-blind trial with attrition and adherence concerns).
Lüftner et al. (2018) [24]	A dermocosmetic kit was associated with less worsening of skin toxicity during chemotherapy with or without radiotherapy and was well tolerated.	Moderate-high (non-randomized observational design with exposure misclassification, subjective assessment, and industry sponsorship).
Berger et al. (2018) [25]	A structured dermocosmetic regimen during breast radiotherapy was well tolerated and associated with fewer early skin reactions and better perceived benefit, without proving efficacy against no skin care.	Moderate–high (observational study with industry involvement, no randomized control, and subjective endpoints).
Dika et al. (2017) [26]	Early cutaneous adverse events were common but mostly mild, and severe reactions were rare and generally responsive to conventional treatment.	Low-moderate (prospective study with small sample size, no control group, and outcome detection concerns).
Shi et al. (2016) [27]	Lichenoid eruptions were an identifiable immune-related toxicity pattern and were usually manageable with topical corticosteroids, with rare discontinuation.	Moderate-high (ROBINS-I: retrospective series with confounding and selection bias).
Bitar et al. (2016) [28]	BTK inhibitors were associated with frequent low-grade hair and nail toxicities that had relevant quality-of-life impact.	Low-moderate (ROBINS-I prospective trial substudy with small simple size, no control group and outcome detection concerns).
Ren et al. (2015) [29]	Prophylactic 10% urea cream reduced the incidence and severity of sorafenib-related HFSR, delayed onset, and improved quality of life without affecting efficacy.	Low-moderate (RoB-2: randomized trial with lack of blinding as the main concern).

BTK, bruton tyrosine kinase; EGFR, epidermal growth factor receptor; GRADE, grading of recommendations assessment, development and evaluation; HFSR, hand–foot skin reaction; MEK, mitogen-activated protein kinase; RoB-2, revised Cochrane risk of bias tool for randomized trials; ROBINS-I, risk of bias in non-randomized studies of interventions.

**Table 3:** Main findings of the included reviews and guidelines, and risk of bias assessments and GRADE evaluation.

Reference (Year)	Main Finding	Risk of Bias
Eshaq et al. (2025) [30]	CAEs were described as the most common immune-related adverse events, with mechanistic links involving immune activation and vitiligo as a possible response marker.	High (narrative review; critical appraisal identified important limitations in search transparency, study selection, and quantitative synthesis).
Madan et al. (2024) [31]	Psoriasis may improve or worsen with targeted therapies and immune checkpoint inhibitors, and early dermato-oncology collaboration may help reduce treatment interruption.	Moderate-high (systematic review based largely on case reports and case series, with ROBIS indicating concerns in the review process and AMSTAR 2 suggesting only moderate methodological quality).
Haynes et al. (2023) [32]	EGFR-targeted therapies were associated with predictable and severe skin reactions, and clinicopathologic correlation was considered important for diagnosis and management.	Moderate (narrative review; critical appraisal identified moderate concerns related to selective literature coverage and lack of systematic search methods).
Emvalomati et al. (2023) [33]	Nail toxicities were common across chemotherapy, targeted therapies, and immune checkpoint inhibitors, and cryotherapy was suggested as a preventive strategy for taxane-related toxicity.	High (narrative review; critical appraisal identified substantial limitations related to non-systematic methods and reliance on lower-level evidence).
Macdonald et al. (2015) [34]	Dermatologic toxicities were common across targeted therapies, and supportive management was considered important to prevent treatment interruption and support multidisciplinary care.	Moderate-high (narrative review; critical appraisal identified relevant concerns regarding selective evidence synthesis and absence of systematic review methods).

AMSTAR, assessing the methodological quality of systematic reviews; EGFR, epidermal growth factor receptor; GRADE, grading of recommendations assessment, development and evaluation; ROBIS, risk of bias in systematic reviews.

**Table 4:** GRADE assessment of the certainty of evidence for the overall findings.

**Overall finding 1:** Dermatologic toxicities are common across cancer therapies but are usually mild to moderate and generally manageable with supportive treatment.			
**Contributing studies**	**Summary of evidence**	**GRADE**	**Main reasons for rating**
Kale et al. (2025) [18]; Friedland et al. (2024) [19]; Anoop et al. (2021) [21]; Keiser et al. (2021) [22]; Dika et al. (2017) [26]; Haynes et al. (2023) [32]; Macdonald et al. (2015) [34]	Across observational studies and secondary sources, dermatologic toxicities were frequent in patients receiving chemotherapy, EGFR-targeted therapy, MEK inhibitors, and immune checkpoint inhibitors. Most reported events were non-severe and were managed with topical or oral supportive measures, with limited treatment discontinuation.	⨁⨁◯◯ Low	Downgraded for predominance of observational evidence, risk of bias, heterogeneity across cancer treatments and toxicity definitions, and indirectness from narrative reviews.
**Overall finding 2:** MEK inhibitors and EGFR-targeted therapies are associated with recognizable patterns of cutaneous toxicity			
**Contributing studies**	**Summary of evidence**	**GRADE**	**Main reasons for rating**
Kale et al. (2025) [18]; Friedland et al. (2024) [19]; Dávila Osorio et al. (2021) [20]; Haynes et al. (2023) [32]	EGFR inhibitors were mainly associated with acneiform eruptions and eczema, whereas pediatric MEK inhibitor therapy was frequently associated with xerosis, dermatitis, paronychia, hair changes, and acneiform manifestations.	⨁⨁◯◯ Low	Downgraded for observational design, confounding, selection bias, and limited direct comparative evidence.
**Overall finding 3:** Immune checkpoint inhibitor-related cutaneous toxicities appear frequent, with eczematous, morbilliform, and lichenoid patterns that are usually manageable			
**Contributing studies**	**Summary of evidence**	**GRADE**	**Main reasons for rating**
Keiser et al. (2021) [22]; Shi et al. (2016) [27]; Eshaq et al. (2025) [30]	Primary and secondary evidence consistently described immune-related skin toxicities as common, with most cases improving with conventional dermatologic treatment and only rare discontinuation of anticancer therapy.	⨁⨁◯◯ Low	Downgraded for retrospective and narrative evidence, risk of bias, and imprecision.
**Overall finding 4:** Prophylactic urea-based interventions may reduce sorafenib-related hand–foot skin reaction severity or delay onset, but evidence is not fully consistent.			
**Contributing studies**	**Summary of evidence**	**GRADE**	**Main reasons for rating**
Ren et al. (2015) [29]; Lee et al. (2020) [23]	One randomized trial reported reduced incidence and severity of sorafenib-related HFSR with 10% urea cream, whereas another found no reduction in overall 12-week incidence with 20% urea cream but did show benefit for early grade II or higher HFSR and quality of life.	⨁⨁⨁◯ Moderate	Randomized evidence supports the finding, but certainty was downgraded for inconsistency between trials and some risk-of-bias concerns.
**Overall finding 5:** Dermocosmetic and supportive skin-care strategies may improve tolerability and patient-reported benefit during cancer treatment, although efficacy evidence remains limited.			
**Contributing studies**	**Summary of evidence**	**GRADE**	**Main reasons for rating**
Lüftner et al. (2018) [24]; Berger et al. (2018) [25]; Macdonald et al. (2015) [34]	Supportive dermocosmetic interventions were generally well tolerated and associated with less worsening of symptoms or fewer early skin reactions, but studies were mostly non-randomized and relied on subjective outcomes.	⨁◯◯◯ Very low	Downgraded for serious risk of bias, non-randomized designs, subjective endpoints, and imprecision
**Overall finding 6:** Hair and nail toxicities may have relevant quality-of-life impact, but evidence for prevention and management is limited			
**Contributing studies**	**Summary of evidence**	**GRADE**	**Main reasons for rating**
Bitar et al. (2016) [28]; Emvalomati et al. (2023) [33]	Hair and nail toxicities were described as frequent but often low-grade; however, they may affect quality of life. Preventive approaches such as cryotherapy were suggested, but support comes mainly from low-level evidence.	⨁◯◯◯ Very low	Downgraded for limited primary evidence, reliance on narrative review data, and indirectness regarding management effectiveness
**Overall finding 7:** Early multidisciplinary supportive care, including dermato-oncology and pharmaceutical care input, may help reduce treatment interruption, but direct evidence remains sparse.			
**Contributing studies**	**Summary of evidence**	**GRADE**	**Main reasons for rating**
Madan et al. (2024) [31]; Macdonald et al. (2015) [34]; relevant contextual support from primary studies [18,22,26]	Secondary sources and contextual interpretation of primary studies suggest that structured supportive care may improve tolerability and continuity of anticancer treatment, but direct interventional evidence specifically evaluating multidisciplinary or pharmaceutical care models is limited.	⨁◯◯◯ Very low	Downgraded for indirectness, reliance on secondary sources, and lack of direct controlled studies of multidisciplinary/pharmaceutical care interventions.

Abbreviations: EGFR, epidermal growth factor receptor; HFSR, hand–foot skin reaction; MEK, mitogen-activated protein kinase kinase.

**Table 5:** Main dermatologic adverse events and preventive and therapeutic strategies.

Adverse Event	Typical Severity and Impact	Prevention		Treatment		Reported Effects on Outcomes
Acneiform rash/folliculitis	Usually mild to moderate; may be painful, visible, and adherence-limiting	† Gentle cleansing, avoid irritants and occlusive cosmetics, daily emollients, sunscreen, early education	‡ Oral tetracycline prophylaxis in higher-risk patients	‡ Continue skin-barrier care, avoid sun and friction	† Topical corticosteroids, topical antibiotics when indicated, oral doxycycline or minocycline; systemic corticosteroids in selected severe cases	Often reduces symptom burden and need for dose modification; improves tolerability
Xerosis/pruritus	Very common; often low grade but persistent and distressing	‡ Soap substitutes, lukewarm showers, fragrance-free moisturizers, regular emollient use	‡ Limited role; sometimes topical anti-inflammatory prophylaxis in high-risk inflammatory dermatoses	† Barrier repair, avoidance of irritants, cooling measures, scratch prevention	† Topical corticosteroids for eczematous inflammation, oral antihistamines, pramoxine or other antipruritics	Can improve comfort, sleep, and adherence when treated early
Hand–foot syndrome	Ranges from erythema and dysesthesia to painful hyperkeratosis and functional limitation	† Reduce friction and heat, protective footwear/gloves, regular skin inspection, urea-based creams	‡ In some settings, prophylactic pyridoxine is used, though evidence is inconsistent	† Activity modification, rest, cooling, avoid pressure and trauma	† Topical corticosteroids, keratolytics such as urea/salicylic acid, analgesics, dose reduction or interruption if severe	May reduce severity and delays; improves daily functioning when proactively managed
Radiation dermatitis	Cumulative, from mild erythema to moist desquamation or ulceration	† Gentle cleansing, moisturization, friction avoidance, sun protection, structured nursing education	† Topical corticosteroids may be used prophylactically in some protocols	† Non-adherent dressings, wound care, infection surveillance, avoid trauma	† Topical corticosteroids, antiseptics or antibiotics if infected, advanced dressings for moist desquamation	May reduce early worsening and improve tolerance of radiotherapy
Maculopapular/immune-related rash	Usually mild to moderate, but may become extensive or symptomatic	† Moisturizers, photoprotection, early reporting of lesions	‡ No standard routine prophylaxis; sometimes topical anti-inflammatory support in selected patients	‡ Avoid irritants, maintain skin hydration, monitor progression	† Topical corticosteroids, oral antihistamines, systemic corticosteroids for more severe cases, temporary treatment interruption if needed	Improves symptom control and may prevent escalation or treatment discontinuation
Photosensitivity	Often preventable; may be sudden and symptomatic even after brief exposure	†Strict photoprotection, broad-spectrum sunscreen, hats, protective clothing, patient education	‡ No routine systemic prophylaxis	† Cool compresses, soothing emollients, avoid further UV exposure	† Topical corticosteroids if inflamed, analgesics as needed	Strongly dependent on preventive counselling; can avoid recurrence
Pigmentary changes/vitiligo-like lesions	Usually not medically severe, but highly visible and body-image affecting	† Photoprotection, avoid trauma and irritation	‡ No established routine prophylaxis	‡ Cosmetic camouflage, reassurance, monitoring	† Topical corticosteroids, calcineurin inhibitors, selected phototherapy for vitiligo-like lesions	Main effect is psychosocial; may affect body image more than physical function
Alopecia/hair disorders	Often reversible but emotionally significant; body-image impact can be substantial	† Gentle hair care, scalp protection, scalp cooling when appropriate	† Scalp cooling in selected chemotherapy regimens	‡ Wigs, head coverings, cosmetic support, counselling	‡ Topical or oral minoxidil in selected cases after treatment or when appropriate	Improves self-image and treatment acceptability; may reduce distress
Paronychia/nail toxicity	Often chronic, painful, and functionally limiting	† Nail care education, avoid trauma, gloves for wet work, keep nails short	‡ No routine pharmacological prophylaxis	‡ Reduce pressure and trauma, warm soaks, local hygiene	† Topical antiseptics, topical corticosteroids, topical antibiotics; systemic antibiotics if infected	Can reduce infection risk and preserve function
Severe cutaneous adverse reactions	Rare but potentially life-threatening	‡ Early recognition and patient education about warning signs	‡ None routinely	‡ Immediate discontinuation, urgent evaluation, supportive care	‡ Systemic corticosteroids or specialist-directed therapy depending on syndrome	Critical for safety; may prevent progression and complications

Note. † Recommendations supported by included primary studies; ‡ recommendations drawn from existing guidelines or consensus statements and used to supplement primary evidence where direct data were limited.

## Data Availability

No new data were created or analyzed in this study.

## Supplementary Materials

The following supporting information can be downloaded at: https://www.mdpi.com/article/10.3390/healthcare14162514/s1, Supplementary File S.1—Complete PRISMA checklist; Supplementary File S.2—Table with characteristics of the included studies, including study design, population, intervention, comparators, and outcomes.

## Abbreviations

The following abbreviations are used in this manuscript:

AMSTAR 2	A MeaSurement Tool to Assess systematic Reviews 2
BTK	Bruton tyrosine kinase
CAE	Cutaneous adverse event
EGFR	Epidermal growth factor receptor
GRADE	Grading of Recommendations Assessment, Development and Evaluation
HFSR	Hand–foot skin reaction
ICI	Immune checkpoint inhibitor
MEK	Mitogen-activated protein kinase
MeSH	Medical Subject Headings
PD-1	Programmed cell death protein 1
PD-L1	Programmed death-ligand 1
PICO(R)	Population, Intervention, Comparison, Outcome (Context)
PRISMA	Preferred Reporting Items for Systematic Reviews and Meta-Analyses
PROSPERO	International Prospective Register of Systematic Reviews
RoB 2	Revised Cochrane Risk of Bias tool for randomized trials
ROBINS-I	Risk Of Bias In Non-randomized Studies of Interventions
ROBIS	Risk Of Bias In Systematic reviews
SWiM	Synthesis Without Meta-analysis
TKI	Tyrosine kinase inhibitor

## References

[1] Bray F., Laversanne M., Sung H., Ferlay J., Siegel R.L., Soerjomataram I., Jemal A. (2024). Global cancer statistics 2022: GLOBOCAN estimates of incidence and mortality worldwide for 36 cancers in 185 countries. CA Cancer J. Clin..

[2] Lacouture M.E., Sibaud V., Gerber P.A., van den Hurk C., Fernández-Peñas P., Santini D., Jahn F., Jordan K. (2021). Prevention and management of dermatological toxicities related to anticancer agents: ESMO Clinical Practice Guidelines. Ann. Oncol..

[3] Rosen A.C., Case E.C., Dusza S.W., Balagula Y., Gordon J., West D.P., Lacouture M.E. (2013). Impact of dermatologic adverse events on quality of life in 283 cancer patients: A questionnaire study in a dermatology referral clinic. Am. J. Clin. Dermatol..

[4] Almeida V., Pires D., Silva M., Teixeira M., Teixeira R.J., Louro A., Dinis M.A.P., Ferreira M., Teixeira A. (2023). Dermatological Side Effects of Cancer Treatment: Psychosocial Implications—A Systematic Review of the Literature. Healthcare.

[5] Silva D., Gomes A., Lobo J.M.S., Almeida V., Almeida I.F. (2020). Management of skin adverse reactions in oncology. J. Oncol. Pharm. Pract..

[6] Duarte N.C., Barbosa C.R., Tavares M.G.R., Dias L.P., Souza R.N., Moriel P. (2019). Clinical oncology pharmacist: Effective contribution to patient safety. J. Oncol. Pharm. Pract..

[7] Xiang H.W., Shen Y.Y., Wang J.X., Zhao Y., Yi B.W., Zhao N. (2025). Impact of pharmacist-led interventions on oncology drug therapy outcomes: A systematic review and meta-analysis. Int. J. Pharm. Pract..

[8] Mendes D., Rigueiro G., Silva R.S., Penedones A., Alves C., Sousa G., Batel-Marques F. (2020). Intensive safety monitoring program of antineoplastic medicines: A pilot study in a Portuguese oncology hospital. J. Oncol. Pharm. Pract..

[9] Fernandes J.P., Advinha A.M., Oliveira-Martins S. (2025). Pharmaceutical consultation on patients receiving oral antineoplastic agents: A systematic review. Eur. J. Hosp. Pharm..

[10] Teixeira A., Teixeira M., Herdeiro M.T., Vasconcelos V., Correia R., Bahia M.F., Almeida I.F., Vidal D.G., Pedrosa e Sousa H.F., Dinis M.A.P. (2021). Knowledge and Practices of Community Pharmacists in Topical Dermatological Treatments. Int. J. Environ. Res. Public Health.

[11] Faller H., Schuler M., Richard M., Heckl U., Weis J., Küffner R. (2013). Effects of psycho oncologic interventions on emotional distress and quality of life in adult patients with cancer: Systematic review and meta analysis. J. Clin. Oncol..

[12] White K., Roydhouse J.K., Scott K.M. (2011). Psychosocial Impact of Cutaneous Toxicities Associated with Epidermal Growth Factor Receptor-Inhibitor Treatment. Clin. J. Oncol. Nurs..

[13] Charles C., Bungener C., Razavi D., Mateus C., Routier E., Lanoy E., Verschoore M., Robert C., Dauchy S. (2016). Impact of Dermatologic Adverse Events Induced by Targeted Therapies on Quality of Life. Crit. Rev. Oncol. Hematol..

[14] Chan A., Cameron M.C., Garden B., Boers-Doets C.B., Schindler K., Epstein J.B., Choi J., Beamer L., Roeland E., Russi E.G. (2015). A Systematic Review of Patient-Reported Outcome Instruments of Dermatologic Adverse Events Associated with Targeted Cancer Therapies. Support. Care Cancer.

[15] Arıkan F., Kartöz F., Karakuş Z., Altınışık M. (2024). Body image and social appearance anxiety in patients with cancer undergoing radiotherapy: A cross sectional study. BMC Psychol..

[16] Dunne S., Semple C., Fitch M. (2022). Editorial: Body image following cancer treatment. Front. Psychol..

[17] Page M.J., McKenzie J.E., Bossuyt P.M., Boutron I., Hoffmann T.C., Mulrow C.D., Shamseer L., Tetzlaff J.M., Akl E.A., Brennan S.E. (2021). The PRISMA 2020 statement: An updated guideline for reporting systematic reviews. BMJ.

[18] Kale I.P., McCauley E.M., Patel A.B. (2025). Cutaneous adverse effects of combination epidermal growth factor receptor inhibitor and immune checkpoint inhibitor cancer therapy. Support. Care Cancer.

[19] Friedland R., Glick M., Amitay-Laish I., Toledano H. (2024). Cutaneous Reactions in Pediatric Patients Treated with MEK Inhibitors: A Retrospective Single-Center Study. Dermatology.

[20] Dávila Osorio V.L., Vicente M.A., Baselga E., Salvador H., Cruz O., Prat C. (2021). Adverse cutaneous effects of mitogen-activated protein kinase inhibitors in children. Pediatr. Dermatol..

[21] Anoop T.M., Rona Joseph J., Min P.N., Pranab K.P., Gopan G., Chacko S. (2021). Cutaneous Toxicities in Breast Cancer Patients Receiving Chemotherapy and Targeted Agents––An Observational Clinical Study. Clin. Breast Cancer.

[22] Keiser M.F., Patel A.B., Altan M. (2021). Cutaneous Toxicities in Lung Cancer Patients on Immune Checkpoint Inhibitor Therapy. Clin. Lung Cancer.

[23] Lee Y.S., Jung Y.K., Kim J.H., Cho S.B., Kim D.Y., Kim M.Y., Kim H.J., Seo Y.S., Yoon K.T., Hong Y.M. (2020). Effect of urea cream on sorafenib-associated hand-foot skin reaction in patients with hepatocellular carcinoma: A multicenter, randomised, double-blind controlled study. Eur. J. Cancer.

[24] Lüftner D., Dell’Acqua V., Selle F., Khalil A., Leonardi M.C., Tomás A.D.L.T., Shenouda G., Fernandez J.R., Orecchia R., Moyal D. (2018). Evaluation of supportive and barrier-protective skin care products in the daily prevention and treatment of cutaneous toxicity during systemic chemotherapy. Onco Targets Ther..

[25] Berger A., Regueiro C., Hijal T., Pasquier D., De La Fuente C., Le Tinier F., Coche-Dequeant B., Lartigau E., Moyal D., Seité S. (2018). Interest of Supportive and Barrier Protective Skin Care Products in the Daily Prevention and Treatment of Cutaneous Toxicity during Radiotherapy for Breast Cancer. Breast Cancer.

[26] Dika E., Ravaioli G.M., Fanti P.A., Piraccini B.M., Lambertini M., Chessa M.A., Baraldi C., Ribero S., Andrea A., Melotti B. (2017). Cutaneous adverse effects during ipilimumab treatment for metastatic melanoma: A prospective study. Eur. J. Dermatol..

[27] Shi V.J., Rodic N., Gettinger S., Leventhal J.S., Neckman J.P., Girardi M., Bosenberg M., Choi J.N. (2016). Clinical and Histologic Features of Lichenoid Mucocutaneous Eruptions Due to Anti-Programmed Cell Death 1 and Anti-Programmed Cell Death Ligand 1 Immunotherapy. JAMA Dermatol..

[28] Bitar C., Farooqui M.Z.H., Valdez J., Saba N.S., Soto S., Bray A., Marti G., Wiestner A., Cowen E.W. (2016). Hair and Nail Changes during Long-term Therapy with Ibrutinib for Chronic Lymphocytic Leukemia. JAMA Dermatol..

[29] Ren Z., Zhu K., Kang H., Lu M., Qu Z., Lu L., Song T., Zhou W., Wang H., Yang W. (2015). Randomized Controlled Trial of the Prophylactic Effect of Urea-Based Cream on Sorafenib-Associated Hand-Foot Skin Reactions in Patients with Advanced Hepatocellular Carcinoma. J. Clin. Oncol..

[30] Eshaq A.M., Flanagan T.W., Ba Abbad A.A., Makarem Z.A.A., Bokir M.S., Alasheq A.K., Al Asheikh S.A., Almashhor A.M., Binyamani F., Al-Amoudi W.A. (2024). Immune Checkpoint Inhibitor-Associated Cutaneous Adverse Events: Mechanisms of Occurrence. Int. J. Mol. Sci..

[31] Madan V., Ortiz-López L.I., Sakunchotpanit G., Chen R., Nayudu K., Nambudiri V.E. (2024). Psoriasis in the Era of Targeted Cancer Therapeutics: A Systematic Review on De Novo and Pre-existing Psoriasis in Oncologic Patients Treated with Emerging Anti-neoplastic Agents. Dermatol. Ther..

[32] Haynes D., Morgan E.E., Chu E.Y. (2023). Cutaneous adverse reactions resulting from targeted cancer therapies: Histopathologic and clinical findings. Hum. Pathol..

[33] Emvalomati A., Oflidou V., Papageorgiou C., Kemanetzi C., Giannouli M., Kalloniati E., Efthymiadis K., Koukoutzeli C., Timotheadou E., Trigoni A. (2023). Narrative Review of Drug-Associated Nail Toxicities in Oncologic Patients. Dermatol. Pract. Concept..

[34] Macdonald J.B., Macdonald B., Golitz L.E., LoRusso P., Sekulic A. (2015). Cutaneous adverse effects of targeted therapies: Part I: Inhibitors of the cellular membrane. J. Am. Acad. Dermatol..

[35] Attili I., Tosti G., Pepe F., Passaro A., de Marinis F. (2025). Optimizing management of cutaneous toxicities from amivantamab in first-line non-small cell lung cancer: A clinical perspective. Crit. Rev. Oncol. Hematol..

[36] Vaez-Gharamaleki Y., Akbarzadeh M.A., Jadidi-Niaragh F., Mahmoodpoor A., Sanaie S., Hosseini M.S. (2025). Dermatologic toxicities related to cancer immunotherapy. Toxicol. Rep..

[37] Peuvrel L., Dréno B. (2014). Dermatological toxicity associated with targeted therapies in cancer: Optimal management. Am. J. Clin. Dermatol..

[38] Zhou S., Kishi N., Alerasool P., Rohs N.C. (2024). Adverse Event Profile of Epidermal Growth Factor Receptor Tyrosine Kinase Inhibitors for Non-small Cell Lung Cancer: An Updated Meta-analysis. Target. Oncol..

[39] Lacouture M.E., Mitchell E.P., Piperdi B., Pillai M.V., Shearer H., Iannotti N., Xu F., Yassine M. (2010). Skin toxicity evaluation protocol with panitumumab (STEPP), a phase II, open-label, randomized trial evaluating the impact of a pre-Emptive Skin treatment regimen on skin toxicities and quality of life in patients with metastatic colorectal cancer. J. Clin. Oncol..

[40] Geisler A.N., Phillips G.S., Barrios D.M., Wu J., Leung D.Y.M., Moy A.P., Kern J.A., Lacouture M.E. (2020). Immune checkpoint inhibitor-related dermatologic adverse events. J. Am. Acad. Dermatol..

[41] Ladwa R., Fogarty G., Chen P., Grewal G., McCormack C., Mar V., Kerob D., Khosrotehrani K. (2024). Management of Skin Toxicities in Cancer Treatment: An Australian/New Zealand Perspective. Cancers.

[42] Du Y., Wu W., Chen M., Dong Z., Wang F. (2023). Cutaneous Adverse Events and Cancer Survival Prognosis with Immune Checkpoint Inhibitor Treatment: A Systematic Review and Meta-Analysis. JAMA Dermatol..

[43] Ra H.S., Shin S.J., Kim J.H., Lim H., Cho B.C., Roh M.R. (2013). The impact of dermatological toxicities of anti-cancer therapy on the dermatological quality of life of cancer patients. J. Eur. Acad. Dermatol. Venereol..

[44] Hoheisel S., Roggero J., Mangana J., Dummer R., Kraehenbuehl L., Ramelyte E. (2025). Cutaneous adverse events of pan-RAF inhibitor naporafenib (LXH254) containing drug combinations in NRAS-mutant metastatic melanoma. EJC Ski. Cancer.

[45] Wongkraisri C., Chusuwanrak K., Laocharoenkeat A., Chularojanamontri L., Nimmannit A., Ithimakin S. (2025). Randomized controlled trial on the efficacy of topical urea-based cream in preventing capecitabine-associated hand-foot syndrome. BMC Cancer.

[46] Lacouture M.E., Anadkat M., Jatoi A., Garawin T., Bohac C., Mitchell E. (2018). Dermatologic Toxicity Occurring during Anti-EGFR Monoclonal Inhibitor Therapy in Patients with Metastatic Colorectal Cancer: A Systematic Review. Clin. Color. Cancer.

[47] Yagasaki K., Komatsu H., Soejima K., Naoki K., Kawada I., Yasuda H., Hamamoto Y. (2018). Targeted Therapy-induced Facial Skin Toxicities: Impact on Quality of Life in Cancer Patients. Asia Pac. J. Oncol. Nurs..

[48] Dreno B., Khosrotehrani K., De Barros Silva G., Wolf J.R., Kerob D., Trombetta M., Atenguena E., Dielenseger P., Pan M., Scotte F. (2023). The role of dermocosmetics in the management of cancer-related skin toxicities: International expert consensus. Support. Care Cancer.

[49] Urakawa R., Hashimoto S., Hirohata H., Sakai K., Matsuura K., Ito Y., Tarutani M., Kubota K., Ueda M., Uejima E. (2021). Skin disorder management in oral anticancer drugs by collaboration of hospital pharmacists and community pharmacists. Support. Care Cancer.

[50] Alabbasi A.K., Green M.S., Brammli-Greenberg S., Haj M.B., Preis M., Schwartz N., Lavon O., Klang S., Cohen S. (2026). Role of a Designated Pharmacist in Reducing Adverse Drug Reaction Rates and Preventing Potential Medication Errors in Hematology-Oncology: A Randomized Controlled Trial. JCO Oncol. Pract..

[51] Rudin C.M., Liu W., Desai A., Karrison T., Jiang X., Janisch L., Das S., Ramirez J., Poonkuzhali B., Schuetz E. (2008). Pharmacogenomic and pharmacokinetic determinants of erlotinib toxicity. J. Clin. Oncol..

