# Supplementary material for: Multidisciplinary Strategies to Manage Treatment-Related Dermatologic Toxicity and Improve Quality of Life in Cancer Patients: A Narrative Review
Source: Healthcare (Basel). 2026 Aug 12;14(16):2514. doi: 10.3390/healthcare14162514 (PMC13512126; doi:10.3390/healthcare14162514)
Supplement: Supplementary file 1 [file healthcare-14-02514-s001.zip › Supplementary File S.2.pdf]

Supplementary File S.2. Characteristics of the included studies.

Reference	Study characteristics	Population	Intervention	Comparators	Outcomes
Kale et al. (2025)	Retrospective chart review; single-center (MD Anderson); 2015-2024; Dermatological diagnosis.	N= 44 (median age 69y [41-94]; 62% M)<br>70% Squamous cell carcinoma of the head and neck III/IV and 30% colon adenocarcinoma IV.	EGFRI+ICI: cetuximab-pembrolizumab (72%; med 86d), cetuximab-nivolumab (28%; med 220d).	None (descriptive; EGFRI vs ICI attribution).	**AEs:** 59% patients (26/44) had 42 CAEs (mostly mild-moderate).<br>Acneiform 33% (13), eczema 18% (7), paronychia 10% (4), pyogenic granuloma 5% (2), pruritus/xerosis/lichenoid 5% each (2).<br>Attribution: EGFRI 62%, ICI 12%, either/both 24%;<br>Onset cetux-pembro 21d/nivo 70d<br>Duration 87d (57% >disc 74d med)<br>**Treatment:** topical antibiotic 60%, oral antibiotic 36%, topical CS 33%<br>**Outcomes:** Discontinuation 2% (1 pt), dose reduction 2% (1); Stop reasons: progression 77%, toxicity 11%.
Eshaq et al. (2025)	Narrative review; mech pathophys ICI CAE; 2024 pub; no orig data;	Cancer pts (melanoma/NSCLC/TNBC/renal/GI/endometrial)	ICI: anti-CTLA4 (ipi)/PD1 (nivo/pembro)/PDL1 (atezo etc); mono/combo.	CTLA4 (early/lymph node) vs PD1 (late/periph); mono vs combo (↑inc 5-72%).	**AEs:** CAE inc 30-60% (CTLA4 44-59%>PD1 34-42%>PDL1 20%)<br>Rash/prur/vitiligo/morbil/lichen/psorias/eczema/BP<br>**Treatment:** top CS/AH/emoll/antiIL6/RTX/ IVIG/aprem.
Friedland et al. (2024)	Retrospective cohort; single-center (Schneider Children's Med Ctr); 2016-2023; all MEKi indications; baseline dermatological evaluation; Follow up ≥6m; derm visits tracked.	N= 33 pediatric (mean age 8y [0.46-20]; 67%F.<br>NF1 42%; low-grade glioma 48%, plexiform NF 30%, high-grade glioma 12%	MEKi: trametinib (21; 0.025-0.032 mg/kg/d; ±dabrafenib/sirolimus); selumetinib (8; BSA-based bid).	Trametinib (90% CAE) vs selumetinib (50%) vs tram+dabraf (34%).	**AEs:** 76% CAE (25/33; onset 2.2m); xerosis 80% (G1-2), dermatitis 76% (G2> G1; atop no corr), paronychia 68% (G1-3; toe malalign↑sev), hair heterochromia 60% (G1), acne 36% (adolesc G1-2), alopecia 36% (G1-2), folliculitis 36% (G1-2), cheilitis 24% (G1); rare EM/urticaria/panniculitis; 237 derm visits (mean5); disc 12% (4 tram; G3 paro/EM/derm)<br>**Treatment:** emoll/top CS/CNI/antibiotic/doxy/soaks (partial resp flares).<br>**Outcomes:** dose red partial.
Madan et al. (2024)	Systematic review; PubMed 2008-2023 ("psoriasis" AND "cancer treatment"); de novo/exacerbation psoriasis targeted/ICI.	N=1363 Onc pts (lung/colon/breast/panc/renal/hepatocell/CML/melanoma) w/ pre-exist/de novo psoriasis	Targeted: EGFRi (erlo/gefi/cetu/pani/osimertinib), BCRABL (imatinib/nilotinib), VEGF (sora/beva/suni), ICI (PD1/PDL1/CTLA4: nivo/pembro/ipi).	De novo vs exacerb; EGFRi improv>induce; ICI exacerb 4x>de novo; TKI disc rare.	**AEs:** Psoriasis assoc EGFRi (improv 70-90% cases; rare induce/exac); BCRABL (induce/exac imatinib/nilotinib; improv some); VEGF (improv sora/beva/suni; rare pustular); ICI psoriasis 3.8% cut tox (exac>de novo.<br>**Treatment:** tx top CS/UV/MTX/apre/secu/ uste/guse).<br>**Outcomes:** BSA>10% adjust; disc 13-23%.
Haynes et al. (2023)	Narrative review organized by target (EGFR/PI3K-AKT/VEGF/FGFR/RAS/BRAF/MEK/ERK/MTK/HH-SMO/CCR4/BTK/Nectin-4); clin-histopath correlation; no primary data.	Oncologic patients on targeted drugs (melanoma/NSCLC/CRC/breast/H&N/bladder/thyroid/etc; BRAF 40%, RAS 30%, EGFR/others).	Targeted kinase inhib: EGFRi (erlot/gefit/osimert etc), PI3K/AKTi (copanlisib/idelalisib/ipatasertib), VEGFi (beva/sorafenib/sunitinib), FGFRi (erdafitinib/pemigatinib), RASi (sotorasib/adagrasib), BRAFi (vem/dabraf/encorafenib),	Intra-class (small-mol vs mAb); combo vs mono; vs chemo.	**AEs:** Acneiform/folliculitis (EGFR/AKT/ERK/VEGF/MEK: neutr suppur fol; acantholysis); HFSR (VEGF/FGFR/MTK/BRAF: hyperk/necrosis); SCC/KA/verrucous (BRAFi 11-30%; sun-prot); alopecia (SMOi 55-66%; MEK); vitiligo/pruritus; granulomatous (BRAFi); interface/spongio/granulom (PI3K/CCR4); PG/SS (BTK/MTK); calcinosis/calciphylaxis (FGFRi); pyogenic gran/angiomas (VEGF); TEN-like/ring mitoses (Nectin-4)<br>**Treatment:** dermat-onco collab.

			MEKi (trametinib/binimetinib), ERKi (ulixertinib), MTKi (imatinib/sorafenib), SMOi (vismodegib/sonidegib), CCR4i (mogamulizumab), BTKi (ibrutinib), Nectin-4i (enfortumab vedotin).		
Emvalomati et al. (2023)	Narrative review; PubMed ≤May2021; clin pres/inc/prev/mgmt; tables chemo/targeted/ICI nail tox.	Onc pts (breast/NSCLC/lung/colorectal/melanoma/renal/thyroid/hematologic).	Chemo (taxanes/anthr/plat/antimetab); targeted (EGFR/MEK/BRAF/mTOR/VEGF/BTK/RET/BCRABL/HER2); ICI (PD1/CTLA4/PDL1).	Chemo (taxanes>anthra); targeted (EGFR parony/pyogran); ICI (psorias/lichenoid G1-2).	**AEs:** Nail tox inc high taxanes 35-89% (onychol/parony/hyperpig/Beau/Mees); EGFR 15% parony; MEK/BRAF photo-onychol; ICI late psorias/onychorhex/lunula eryt.<br>**Treatment:** emoll/CS/antibiotic/cryo/frozen gloves/nail clip/infx ctrl<br>**Outcomes:** QoL impact; res post-disc
Dávila Osorio et al. (2021)	Retrospective observational at single pediatric hospital (Hospital Sant Joan de Déu, Barcelona, Spain); reviewed records Jan 2015-Jun 2019; excluded <3 months follow-up; median follow-up 27 months.	61 children (1-18 y; 51% M, median age 7.7 y trametinib group and 13.2 y selumetinib group).<br>Mainly neurofibromas (primary), gliomas.	Mitogen-activated protein kinase inhibitors: trametinib (n=53), selumetinib (n=8).<br>Pre-treatment dermatology education (sunscreen, emollients, nail care).	None (descriptive; no control group); subgroup trametinib vs selumetinib.	**AEs**: 100% cutaneous AEs (all CTCAE Grade 1-2); 2 discontinued due to severe acne.<br>- Eczema (85% trametinib/25% selumetinib, onset 6.2 months)<br>- Hair abnormalities (83%/62.5%, 5.8months)<br>- Paronychia (70%/37.5%, 5.2mo)<br>- Eyelash trichomegaly (53%/25%)<br>- Folliculitis (45%/25%)<br>- Xerosis (42%/25%)<br>- Acne (40%/62.5%, earliest 2.4mo)<br>- Oral aphthae (37%/25%)
Anoop et al. (2021)	Prospective observational; single-center (Reg Cancer Ctr, India); 2019-2020; clin dx; interventions tracked.	N= 250/720 F breast Ca pts w/ CAE (100%F; mean age 48y; neoadj 23%, adj 36%, pall met 27%, targeted met 14%).	Systemic chemo/targeted: docetaxel/paclitaxel/capecitabine/doxo/epirubicin/cyclophosphamide/5FU/lapatinib/everolimus/tamoxifen (regimens: AC-Doce/FEC-Doce/TC/DDAC-Pacli/Finher etc).	Agents (docetaxel>paclitaxel>capecitabine); settings (neoadj/adj/pall).	**AEs:** Hair 96% (alopecia G2 86%; dox/doce/pacli etc), skin 92% (xerosis 92%; hyperpig 88%; inj site disc 67%; HFS 49% G1-3 docetaxel/capecitab; thrombophleb 16%; extrav 14%; macpap rash/pruritus 10%), nail 34% (melanonychia 34% doce/cap; onychol 17%; Beau 14%; parony 6% cap/lap), mucosa 26% (mucositis G1-3 26% doce/doxo/cyclo); rare SJS/cellulitis/flagellate etc.<br>**Treatment:** emoll/CS/antibiotic/soaks/pyridoxine/minoxidil;<br>**Outcomes:** interrup/recov 2-4w (HFS/mucositis G3); delay 3-5w.
Keiser et al. (2021)	Retrospective chart review; single-center (MD Anderson); 2017-2018; Follow up ≥1y; derm/onc confirm; RECIST v1.1 OS/PFS.	N= 64 w/ CAE (med age 68y [30-96]; 63%M; 73%Cauc; smoke 91%. NSCLC: adeno 67%/SCC 28%; stage IV 70%.	Single-agent ICPI: nivolumab 50%/ pembrolizumab 44%/atezolizumab 6%(med exp 14m)	Rash types (eczema/morbilliform/acneiform); prior derm hist (no corr).	**AEs:** CAE 100% cohort (73 total; onset med 3m [0-46m]; dur 4m).<br>Eczema 28%, morbilliform 13%, acneiform 8%, HFD/prur/psoriasiform/shingles 5%; lichenoid/seb derm/urtic/vitiligo 3%; other 2%; unchar 23%; high-grade 23%; prior derm 44% (14% infl,20% actinic)<br>**Treatment:** top CS 61%/oral AH 25%/oral st 9%; resolv 64%/improv 6%/stable 30%<br>**Outcomes**: dose delay 6%/disc 6% CAE (psorias/eczema/urtic/EM; +1 pneumonitis); mPFS 571d/mOS 845d adv.

Lee et al. (2020)	Multicenter, prospective, randomized, double-blind, controlled trial in 13 Korean centers; QoL used for patient-reported outcome	288 HCC pts randomized (140 placebo, 148 urea); 247 included in per-protocol Hand-Foot Skin reaction (HFSR) analysis (117 placebo, 130 urea); mostly male (~83–86%), median age ~60–62 years	20% urea cream applied to hands/feet twice daily for 12 weeks, started with sorafenib 400 mg bid; standard dose-modification algorithm for HFSR (grade-based reductions/interruptions).	Identical placebo cream applied to hands/feet twice daily for 12 weeks, started with sorafenib 400 mg bid	**AEs:** Cumulative any-grade HFSR lower (NS) with urea (log-rank P=0.247); cumulative grade ≥ II HFSR also lower trend (P=0.394); at 2 weeks, severe HFSR (grade ≥ II) significantly less with urea (13.8% vs 23.9%, P=0.042); overall 12-week any-grade HFSR per-protocol 59.8% placebo vs 50.8% urea (P=0.153); grade III similar.<br>**Outcomes:** sorafenib dose modification and interruption rates, average daily dose, tumor response (ORR ~6%, DCR ~43–44%), and 12-week survival similar between groups; safety profile comparable (HFSR, rash, fatigue, diarrhea >10% in both). HF-QoL total and daily-activity scores significantly better with urea at 12 weeks (total 11.8 vs 19.7, P=0.014);
Lüftner et al. (2018)	Multicenter prospective observational open-label study; 5 centers (Germany, France, Italy, Spain, Canada); ~9-week follow-up during chemotherapy ± radiotherapy; post-hoc analysis by product use intensity.	147 cancer patients (39% breast/ovary, 31% GI, 20% H&N); mean age 59y, 71% female; various chemo regimens (alkylating 80%, antimetabolites 44%) ± concomitant (35%) or sequential RT (15%).	Regular users of 12-product dermocosmetic kit (La Roche-Posay): thermal water, cleansing oil, body balm, shampoo, hand/foot repair creams, face moisturizer/cleanser, SPF50+ sunscreens; patients classified as regular (≥7 products used often/daily, 43%) vs casual users (54%).	Casual users	**AEs:** 31% had skin reaction worsening; significantly less in regular vs casual users (22% vs 39.5%, p=0.029); less erythema (p=0.016)/desquamation (p=0.028) aggravation; concomitant RT strongly associated with worsening (78% vs 7%)<br>**Outcomes:** tolerance ≥89% good/excellent; physicians 100% positive opinion for regular users (vs 92% casual, p=0.009); Patient Benefit Index>1 in 95.5%, higher scores for regular users (3.23 vs 2.8, p=0.049).
Berger et al. (2018)	Multicenter, prospective, observational (real-world); 4 centers (France, Spain, Canada); 6-week follow-up over full RT course; no randomized control; analysis by intensity of use of a predefined dermocosmetic kit.	253 women starting adjuvant/neoadjuvant or exclusive external-beam RT for breast cancer (mostly stage I, post-segmental mastectomy; mean age 60 years; Fitzpatrick I–VI, ~72% fair skin).	Predefined non-pharmaceutical skin-care kit (La Roche-Posay: thermal water, gentle cleansing oil, emollient balm, wound-healing balm, SPF50+ sunscreen) given before first fraction; patients instructed on daily/pro re nata use; later classified as "heavy" (score ≥5) users based on frequency across products.	Low<5) users	**AE's:** Overall, 85% developed at least one skin reaction (mainly radiation dermatitis: 86% of events), mostly grade 1 (93%) and some grade 2 (7%); mean onset ~19 days after RT start. Within first 10 days, first skin reaction occurred in significantly fewer heavy vs low users (8% vs 18%, p=0.031); later reactions numerically less frequent but not statistically different.<br>**Outcomes:** >92% rated tolerance "good–excellent" for each product. Physicians' positive opinion on benefit was higher in heavy vs low users (64–66.6% vs 32%, p<0.0001). Patient Benefit Index >1 in most pts, with trend to higher benefit for heavy users (93%, PBI ~2.9) vs low users (86%, PBI ~2.7; p≈0.095).
Dika et al. (2017)	Prospective cohort; single center (U Bologna Skin Cancer Unit); 2013-2016<br>Dermatological evaluation at baseline + post-each cycle (T1-T4).	N= 41<br>Metastatic melanomas; Excluded prior targeted therapy.	Ipilimumab 3mg/kg IV q3w (up to 4 cycles).	None (prospective descriptive; baseline vs post-tx).	**AEs:** 34.1% cutaneous AEs (all G1-2 except 1 G4 SJS). Onset 3-8w.<br>Rash (7.3%; n=3), folliculitis (7.3%; n=3), mucositis (2.4%; n=1), rosacea (2.4%; n=1), eczema craquelé (2.4%; n=1), acneiform (2.4%; n=1), syringometaplasia mucinosa (2.4%; n=1), SJS (2.4%; n=1 G4), vitiligo (4.9%; n=2 G2); severe pruritus >7/10 (9.8%; n=4), xerosis >7/10 (4.9%; n=2); 2 atypical nevi excised (benign); 1 discontinue (G4 SJS).<br>**Treatment:** Topicals/systemic antibiotic/antifungals/CS/AH/ moisturizers (success except G4). No melanoma recurrence.

Shi et al. (2016)	Retrospective case series; Yale-New Haven Hospital dermatology referrals (2010-2015); chart/biopsy review by 2 dermatopathologists; n=20 consecutive.	N= 20 adults (13M/7F; mean age 64y [46-86]).<br>NSCLC (11), melanoma (6), RCC (3); advanced; 60% prior systemic therapy (3 prior ICI).	Anti-PD-1/PD-L1: nivolumab monotherapy (10), +ipi (4), +bev (1), +erlotinib then alone (1); pembrolizumab (2); atezolizumab alone (1), +carbo/paclitaxel (1).	None; concurrent meds noted (80% lichenoid drugs e.g. statins, ACEi); prior therapies.	**AEs:** Onset mean 4mo (3d-12.8mo).<br>-80% erythematous scaly papules;<br>- variants: hypertrophic LP-like, Grover-like, palmoplantar (10%), oral (20%), genital erosion (5%), inflamed SK (5%); pruritus 75%<br>**Treatment:** Topical CS (90%)<br>**Outcomes:** 25% dose delay; 1/20 discontinue; PFS mean 20.1mo (responders 26.9mo).
Bitar et al. (2016)	Prospective survey phase II trial substudy; single-center (NIH); 2014-2015; 11-q survey; severity Van de Kerkhof 0-3/CTCAE v3; QoL impact.	66 CLL pts (age 55-85y; 65%M).	Ibrutinib (420mg/d long-term).	Fingernail vs toenail; M vs F.	**AEs:** Fingernail brittle 67% (onset med 6.5m [6-12]; G1-2 onychoschizia/rrhexis); toenail 23% (9m [6-15]); hair textural 26% (9m [6-12]; straight/soft/curl)<br>**Outcomes:** discomfort/QoL impact 55%; biotin improv 3/5.
Macdonald et al. (2015)	Narrative review focus mechanisms/incidence/clinical management; evidence levels (IA-IV);.<br>Part I membrane inhibitors (EGFR/KIT/BCR-ABL/antiangio/multikinase).	Oncologic patients on targeted drugs:<br><u>EGFRi:</u> CRC/H&N/NSCLC/breast<br><u>KIT/BCR-ABL:</u> CML/GIST<br><u>Antiangiogenis:</u> RCC/HCC<br><u>Multikinase:</u> RCC/HCC/NSCLC/GIST).	Membrane inhibitors: EGFRi (cetux/panit/erlot/gefit/lapatinib/canertinib/vandetanib), KIT/BCR-ABL (imatinib/nilotinib/dasatinib), anti-VEGF (bev/ranibizumab), multikinase (sorafenib/sunitinib/pazopanib/vandetanib).	Intra-class (mono vs combo); vs chemo; dose-dependency.	<u>EGFRi:</u> **AEs:** papulopustular 75-90% (seborrheic; 1-2w; dose-dep; corr efficacy); xerosis 33%; hair (kinky/trichomegaly/hirsutism/alopecia 17%); mucositis; paronychia 17%; photo; **Treatment:** top CS/antibiotics/tetras/isotret; sun protection.<br><u>KIT/BCR-ABL:</u> **AEs:** edema maj; morbilliform 50% (9w); pigment (hypo/hyper); infl (lichen/psor/pityr rosea/AGEP/DRESS/SJS); alopecia; rev dose-dep.<br><u>Antiangio:</u> **AEs:** mucocut hemorr 20-40%; poor wound heal.<br><u>Multikinase:</u> **AEs:** HFSR 10-63% sorafenib (pain hyperk press sites); infl eruptions 10-60%; hair (alop/depig); genital; eruptive nevi; yellow skin; rev.
Ren et al. (2015)	Multicenter (64 sites), investigator-initiated, randomized, open-label, controlled phase III trial; primary endpoint: 12-week incidence of any-grade HFSR; 2-week assessments up to week 12 + week 14 follow-up; modified HFSR-specific grading; HF-QoL questionnaire.	871 Chinese patients with advanced HCC starting sorafenib 400 mg BID; median age ~52y, 86% male, HBV etiology 77%, ECOG PS 0-1 (96%), prior locoregional therapy 68%; 871 randomized and evaluable for HFSR.	10% urea-based cream (Eucerin) TID to hands/feet + BSC (excluding creams) started day 1 of sorafenib; after HFSR, any cream permitted; sorafenib dose modifications per protocol for HFSR grades.	BSC alone (no creams until HFSR onset)	**AEs:** Significant reduction in any-grade (56% vs 74%) and grade ≥2 HFSR (21% vs 29%); delayed onset (84 vs 34 days); similar other AEs.<br>**Outcomes:** sorafenib dosing compliance, response rates; better HF-QoL (lower symptom/daily activity burden with UBC); elevated AST predicted higher HFSR risk but UBC benefit consistent across subgroups.

AE, adverse event; BRAF, v-raf murine sarcoma viral oncogene homolog B1; BSA, body surface area; CAE, cutaneous adverse event; CS, corticosteroid; AH, anti-histamine; CTCAE, Common Terminology Criteria for Adverse Events; DRESS, drug reaction with eosinophilia and systemic symptoms; EGFR, epidermal growth factor receptor; EGFRi, epidermal growth factor receptor inhibitor; G, grade; HER2, human epidermal growth factor receptor 2; ICI, immune checkpoint inhibitor; IV, intravenous; KIT, tyrosine-protein kinase Kit; M, male; MAPK, mitogen-activated protein kinase; MEK, mitogen-activated protein kinase kinase; mo, months; NSCLC, non-small cell lung cancer; pts, patients; q3w, every 3 weeks; y, years; BCR-ABL, breakpoint cluster region - abelson murine leukemia viral oncogene homolog 1; FGFR, fibroblast growth factor receptor; RAS, rat sarcoma; ERK, extracellular signal-regulated kinase; MTK, map kinase kinase kinase; HH-SMO, hedgehog - smoothened; CCR4, c-c chemokine receptor type 4; BTK, bruton's tyrosine kinase; clin-histopath, clinical-histopathological; derm, dermatology; clin dx, clinical diagnosis; QoL, quality of life; clin pres, clinical presentation; inc, incidence; prev, prevalence; mgmt,management; Onc, oncology; CRC,colorectal cancer; H&N, head and neck cancer; CML, chronic myeloid leukemia; GIST, gastrointestinal stromal tumor ; RCC, renl cell carcinoma ; HCC, hepatocellular carcinoma; TNBC, triple-negative breast cancer; cauc, caucasian; CLL, chronic lymphocytic leukemia; ipi, ipilimumab; bev, bevacizumab; bid, twice a day; dose-dep, dose-dependent; corr efficacy, correlation with efficacy; tx, treatment; rev, reversible; neoadj, neoadjuvant; adj, adjuvant; pall, palliative; prur, pruritus; emoll, emollients; infx ctrl, infection control; apre, apremilast; secu, secukinumab; uste, ustekinumab; guse, guselkumab; HFSR, Hand-Foot Skin reaction; PBI – Patient Benefit Ind
